# Correction: *N*^6^-methyladenosine of HIV-1 RNA regulates viral infection and HIV-1 Gag protein expression

**DOI:** 10.7554/eLife.31482

**Published:** 2017-09-13

**Authors:** Nagaraja Tirumuru, Boxuan Simen Zhao, Wuxun Lu, Zhike Lu, Chuan He, Li Wu

Tirumuru N, Zhao BS, Lu W, Lu Z, He C, Wu L. 2016. N^6^-methyladenosine of HIV-1 RNA regulates viral infection and HIV-1 Gag protein expression. *eLife* **5**:e15528. doi: 10.7554/eLife.15528.

Published 2, July 2016

We would like to issue the following correction to our research article entitled, ‘*N*^6^-methyladenosine of HIV-1 RNA regulates viral infection and HIV-1 Gag protein expression’. In the original version of our article, we noticed two errors in the results of the crosslinking and immunoprecipitation assay combined with RNA-seq (CLIP-seq) experiments performed to identify YTHDF1-3 binding sites on HIV-1 RNA genome (results reported in Figure 1C):

(1) We used a luciferase-expressing reporter HIV-1 in the experiment, but used wild-type (WT) HIV-1_NL4-3_ genome in the sequencing analysis and data presentation due to a miscommunication between two collaborating labs;

(2) YTHDF1-3 bound RNA fragments were over-digested during CLIP-seq sample preparation, resulting in abnormally short reads and large amount of dual-mapping reads to both HIV-1 and human genomes, causing inaccurate representation of actual binding sites of YTHDF1-3.

To more accurately map the binding sites on the HIV-1 RNA genome by YTHDF1-3, we repeated the CLIP-seq experiments using WT HIV-1 strain infecting HeLa cell lines expressing the HIV-1 primary receptor CD4 (HeLa/CD4), and fresh RNase T1 stocks during CLIP-seq sample preparation. In this repeat experiment, we used X4-tropic, replication-competent HIV-1_NL4-3_ to infect HeLa/CD4 cells that overexpressed CD4 and FLAG-tagged YTHDF1-3 proteins. Our previous results also confirmed that YTHDF1–3 significantly inhibited WT HIV-1_NL4-3_ replication in HeLa/CD4 cells (For details, please refer to Figures 1 and 2 of authors’ responses posted on March 27, 2017 on the *eLife* paper site: https://elifesciences.org/articles/15528).

For the CLIP-seq, we sequenced the cDNA library with sufficient depth to obtain precise reads. We obtained a total of 2×10^4^ to 2×10^5^ reads from each sample to cover HIV-1 genome based solely on unique reads specifically mapped to HIV-1_NL4-3_ genome, but not the human genome. We used 16 nucleotides (nt) as a cutoff to filter out the short reads, with all reads analyzed possessing an average size of 30 nt.

Finally, we have removed the old data and uploaded all the updated raw data into GEO under the same accession number GSE85724.

Changes made to correct the error in Fig. 1C and corresponding text and methods sections are listed below:

**Change 1:** The original Fig. 1C has been replaced to report the new CLIP-seq results, showing differences in the exact binding sites of YTHDF proteins.

Original Fig. 1 and legend:

**Figure 1:**
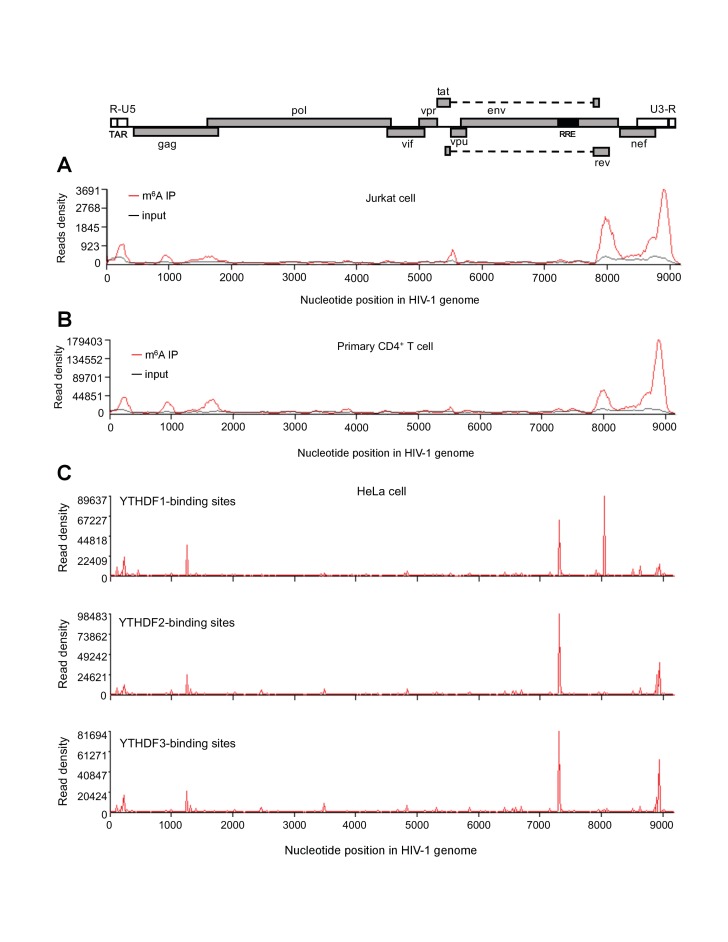
HIV-1 RNA contains m^6^A modifications and YTHDF1–3 proteins bind to m^6^A-modified HIV-1 RNA. (**A**–**B**) The distribution of m^6^A reads from m^6^A-seq mapped to HIV-1 genome (red line) in HIV-1 infected Jurkat cells (**A**) or primary CD4^+^ T-cells (**B**). Baseline signal from the RNA-seq of input samples is shown as a black line. A schematic diagram of HIV-1_NL4-3_ genome is shown above. TAR, transacting response element; RRE, Rev response element. Jurkat cells (**A**) or primary CD4^+^ T-cells (**B**) were infected with HIV-1_NL4-3_ and total RNA was extracted for m^6^A-seq at 72 or 96 hr post-infection (hpi), respectively. (**C**) YTHDF1–3 proteins bind to the HIV-1 gRNA. HeLa cells overexpressing individual FLAG-tagged YTHDF1–3 proteins were infected with HIV-1-Luc/VSV-G at a multiplicity of infection (MOI) of 0.5 for 2 hr. CLIP assay was performed with anti-FLAG. The peaks represent the read density and the HIV-1 genome organization, and corresponding nucleotide positions are shown. All data presented are representative of duplicate samples (n=2) in at least two independent experiments.

New Fig. 1 and legend (only Fig. 1C is changed):

**Figure 2:**
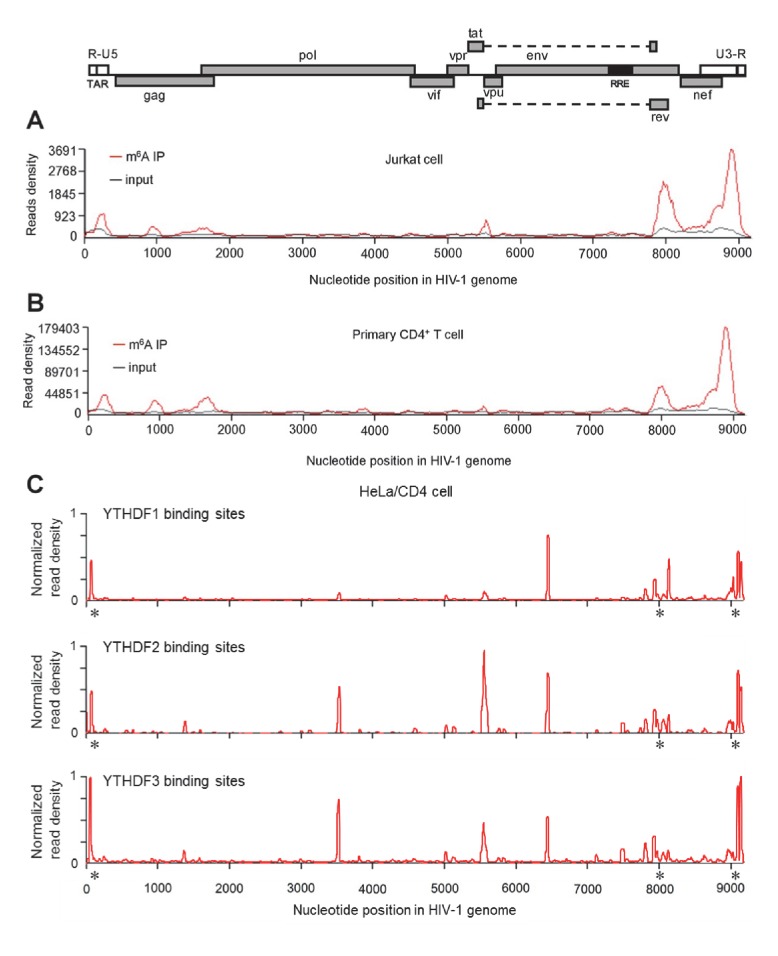
HIV-1 RNA contains m^6^A modifications and YTHDF1–3 proteins bind to m^6^A-modified HIV-1 RNA. (**A**–**B**) The distribution of m^6^A reads from m^6^A-seq mapped to HIV-1 genome (red line) in HIV-1 infected Jurkat cells (**A**) or primary CD4^+^ T-cells (**B**). Baseline signal from the RNA-seq of input samples is shown as a black line. A schematic diagram of HIV-1_NL4-3_ genome is shown above. TAR, transacting response element; RRE, Rev response element. Jurkat cells (**A**) or primary CD4^+^ T-cells (**B**) were infected with HIV-1_NL4-3_ and total RNA was extracted for m^6^A-seq at 72 or 96 hr post-infection (hpi), respectively. (**C**) YTHDF1-3 proteins bind to the HIV-1 gRNA. HeLa/CD4 cells overexpressing FLAG-tagged YTHDF1-3 proteins were infected with HIV-1_NL4-3_ (MOI= 5) for 72 hr and used in CLIP-seq assay to identify their binding sites on HIV-1 gRNA. The distribution of mapped reads (>16 nt) with corresponding nucleotide positions are shown, forming peaks as putative binding positions. Asterisks mark the peak clusters overlapping with identified m^6^A peaks, indicating high-confident YTHDFs binding sites. Read density was normalized to the total number of mapped reads in each sample (YTHDF1: 28438; YTHDF2: 232568; YHTDF3: 124915). The data presented are representative of results from two independent experiments (n=2).

**Change 2:** The main text section entitled “YTHDF1–3 proteins bind to HIV-1 gRNA in infected cells” has been updated to reflect the data changes.

Original main text section:

YTHDF1–3 proteins are reader proteins that specifically bind to m^6^A-methylated cellular RNAs (Wang et al., 2014, 2015). We utilized the crosslinking and immunoprecipitation (CLIP) assay combined with RNA-seq (Hafner et al., 2010; Liu et al., 2015) to map the binding sites of YTHDF1–3 proteins in the HIV-1 genome in infected HeLa cells that overexpressed individual FLAG-tagged YTHDF1–3 proteins (Figure 2A). We identified multiple CLIP peaks of YTHDF1–3 protein-bound HIV-1 RNA, including the transactivation response element (TAR) in the 5’ UTR leader sequence, the *gag* gene, the Rev response element (RRE) in the *env* gene, in the *rev* gene, and the 3’ UTR (Figure 1C). Some YTHDF-binding sites (such in the 3’ and 5’ UTR and the *gag* gene) partially overlap with the identified m^6^A-containing regions in the HIV-1 genome (Figure 1A–C). Of note, we observed a unique high peak within the *rev* gene of HIV-1 RNA bound by YTHDF1 (Figure 1C, top panel), suggesting that YTHDF1 may bind HIV-1 RNA differently from YTHDF2–3. These data demonstrate that YTHDF1–3 proteins bind to m^6^A-modified HIV-1 gRNA during viral infection.

New main text section:

YTHDF1–3 proteins are reader proteins that specifically bind to m^6^A-methylated cellular RNAs (Wang et al., 2014, 2015). We utilized the crosslinking and immunoprecipitation (CLIP) assay combined with RNA-seq (Hafner et al., 2010; Liu et al., 2015) to map the binding sites of YTHDF1–3 proteins in the HIV-1 genome in infected HeLa/CD4 cells that overexpressed individual FLAG-tagged YTHDF1–3 proteins. We observed multiple CLIP peaks from YTHDF1–3 protein-bound HIV-1 RNA, including the transactivation response element (TAR) in the 5’ UTR leader sequence, the *env* gene, the *rev* gene, and the 3’ UTR. Some YTHDF-binding sites (e.g. at the 3’ and 5’ UTR and the *gag* gene) in HIV-1_NL4-3_ infected HeLa/CD4 cells partially overlap with the identified m^6^A-containing regions in the HIV-1 genome in HIV-1_NL4-3_ infected CD4+ Jurkat T-cells or primary CD4+ T-cells (marked by asterisks), indicating high-confident YTHDF1-3 binding sites. Different cell types used in these experiments might contribute to the difference of the m^6^A sites and YTHDFs-bound sites in HIV-1 RNA genome. Overall, these data confirm our previous results that YTHDF1-3 proteins bind to m^6^A-modified HIV-1 genomic RNA during viral infection.

**Change 3:** The CLIP-seq protocol in materials and methods section has been updated to reflect additional details from the repeat experiments.

Original protocol:

We followed the previously reported protocol of the PAR (photoactivatable ribonucleoside-enhanced)-CLIP assay (Hafner et al., 2010) with the following modifications. As HIV-1 infection was inhibited by the addition of 4-thiouridine (data not shown), we omitted that step and performed crosslinking directly. Briefly, six 15-cm plates of HeLa cells stably expressing YTHDF1–3 proteins were seeded at day 1. At day 2, the HeLa cells were infected with the HIV-1-Luc/VSV-G at an MOI of 0.5 for 2 hr, cells were washed to remove cell-free HIV-1. At day 3, the cells were washed once with 10 mL ice-cold PBS. Cell plates were placed on a tray with ice and irradiated, uncovered, with 0.15 J/cm^2^ of 254 nm UV light three times in a Stratalinker 2400 (Stratagene, Santa Clara, CA). Cells were scraped off in PBS and transferred to centrifugation tubes and collected by centrifugation at 500 ×g for 5 min at 4°C. The cell pellets were lysed in 3 volumes of 1% NP40 lysis buffer and incubated on ice for 10 min. The cell lysates were cleared by centrifugation at 13,000 ×g for 15 min at 4°C. Anti-FLAG antibodies were used in the IP as previously described. The recovered RNA samples were further cleaned by RNA Clean & Concentrator (Zymo Research, Irvine, CA) before library construction using the small RNA sample preparation kit (NEB). The first round of RNase T1 digest was carried out at 0.2 U/μl for 15 min and the second round digestion was conducted at 10 U/μl for 8 min.

New protocol:

We followed a previously reported protocol of the PAR (photoactivatable ribonucleoside-enhanced)-CLIP assay (Hafner et al., 2010) with the following modifications. As HIV-1 infection was inhibited by the addition of 4-thiouridine (data not shown), we omitted that step and performed crosslinking directly. Briefly, HeLa/CD4-Y1 cells stably expressing individual YTHDF1-3 proteins were seeded in thirty 15-cm diameter plates one day before HIV-1_NL4-3_ infection. At day 2, the cells were infected with HIV-1_NL4-3_ at a multiplicity of infection (MOI) of 5 and cells were washed to remove cell-free viruses. At 72 hr post-infection, the cells were washed once with 10 mL ice-cold PBS. Uncovered cell plates were placed on a tray with ice and irradiated with 0.15 J/cm^2^ of 254 nm UV light three times in a Stratalinker 2400 (Stratagene, Santa Clara, CA). Cells were scraped off in PBS and transferred to centrifugation tubes and collected by centrifugation at 500 × g for 5 min at 4°C. The cell pellets were lysed in 3 volumes of 1% NP40 lysis buffer and incubated on ice for 10 min. The cell lysates were cleared by centrifugation at 13,000 × g for 15 min at 4°C. Cleared cell lysates were incubated with RNase T1 to a final concentration of 0.2 U/μl, at 22°C for 15 min, immediately put on ice for 5 min to quench. Samples were then centrifuged at 13,000 × g for 10 min at 4 °C and the supernatant was taken. Anti-FLAG M2 magnetic beads (Sigma M8823, 20 μl slurry/15-cm diameter plate) were washed in IP buffer (50 mM HEPES (pH 7.5), 0.3 M KCl, 0.05% NP40) for 5 times and resuspended in cleared cell lysates and incubated at 4 °C overhead rotator for 2 hr. After 2 hr, beads were washed with 1 mL IP buffer for 3 times and beads were changed into a new tube for the final wash and resuspended into 100 μl IP buffer. RNAse T1 was added to the beads at a final concentration of 10 U/μl, incubated at 22°C for 6 min, immediately put on ice for 5 min to quench. Beads were washed with 1 mL high salt buffer (50 mM Tris (pH 7.5), 500 mM KCl, 0.05% NP40) five times, then with 1 mL T4 polynucleotide kinase (PNK) buffer containing 50 mM Tris (pH 7.5), 10 mM MgCl_2_, 50 mM NaCl (without DTT) two times, finally resuspended into 100 μl PNK reaction mix (95 μl commercial PNK buffer and 5 μl T4 PNK (Promega) and incubated at 37°C for 15 min. Then 10 μl PNK and 1.1 μl 10 mM ATP (to final concentration of 100 μM) were added to the mixture and kept at 37°C for another 20 min, followed by washing with 1 mL PNK buffer (without DTT) five times and 1 mL high salt buffer five times. The bound RNA fragments were eluted from the beads by proteinase K digestion twice at 55 °C for 20 and 10 min, respectively. The eluate was further purified using RNA Clean and Concentrator kit (Zymo Research). RNA was used for library generation with NEBNext Small RNA Library Prep kit (NEB). Sequencing was carried out on Illumina HiSeq 4000 according to the manufacturer’s instructions.

For bioinformatics analysis, after removing the adapter sequence, only reads that are longer than 16 bp were kept. The reads were mapped to the reference genomes (both human hg38 and HIV-1_NL4-3_ (GenBank: M19921.2) using Bowtie2. Reads uniquely mapped to HIV-1 genome (not to human genome) were used in the subsequent analysis.

